# Cytological, Physiological, and Transcriptomic Analyses of the Leaf Color Mutant *Yellow Leaf 20* (*yl20*) in Eggplant (*Solanum melongena* L.)

**DOI:** 10.3390/plants13060855

**Published:** 2024-03-15

**Authors:** Bing Li, Jingjing Zhang, Peng Tian, Xiurui Gao, Xue Song, Xiuqing Pan, Yanrong Wu

**Affiliations:** 1 Institute of Cash Crops, Hebei Academy of Agriculture and Forestry Sciences, Shijiazhuang 050051, China; lbhbnky@163.com (B.L.); tianpengtdc@163.com (P.T.); songxue2024@126.com (X.S.); panxiuqing63@126.com (X.P.); 2 Hebei Vegetable Technology Innovation Center, Shijiazhuang 050051, China

**Keywords:** eggplant (*Solanum melongena* L.), transcriptome, leaf color, chlorophyll metabolism, chloroplast development

## Abstract

Leaf color mutants are ideal materials for studying chlorophyll metabolism, chloroplast development, and photosynthesis in plants. We discovered a novel eggplant (*Solanum melongena* L.) mutant *yl20* (yellow leaf 20) that exhibits yellow leaves. In this study, we compared the leaves of the mutant *yl20* and wild type (WT) plants for cytological, physiological, and transcriptomic analyses. The results showed that the mutant *yl20* exhibits abnormal chloroplast ultrastructure, reduced chlorophyll and carotenoid contents, and lower photosynthetic efficiency compared to the WT. Transcriptome data indicated 3267 and 478 differentially expressed genes (DEGs) between WT and *yl20* lines in the cotyledon and euphylla stages, respectively, where most DEGs were downregulated in the *yl20*. Gene Ontology (GO) analysis revealed the “plastid-encoded plastid RNA polymerase complex” and the “chloroplast-related” terms were significantly enriched. Kyoto Encyclopedia of Genes and Genomes (KEGG) analysis demonstrated that the significantly enriched DEGs were involved in flavone and flavonol biosynthesis, porphyrin and chlorophyll metabolism, etc. We speculated that these DEGs involved in significant terms were closely related to the leaf color development of the mutant *yl20*. Our results provide a possible explanation for the altered phenotype of leaf color mutants in eggplant and lay a theoretical foundation for plant breeding.

## 1. Introduction

Leaf color variation arises from the mutations in genes associated with chloroplast development and chlorophyll metabolism, which further influences chlorophyll biosynthesis, photosynthesis, photomorphogenesis, and related signal transduction pathways [1]. The green color of the leaves is primarily attributed to substantial chlorophyll accumulation [2]. Chlorophyll accumulation is regulated by 27 genes encoding 15 enzymes that participate in the chlorophyll biosynthesis pathway [3,4]. Mutation to a cytochrome P-like gene alters the leaf color by affecting the chlorophyllbiosynthesis pathways in *Brassica napus* [5,6]. Transcriptome investigations can increase our understanding and provide novel insights into the mechanisms underlying leaf color formation [7,8]. For instance, a previous study focusing on the chlorophyll biosynthesis pathway reported a downregulation of most differentially expressed genes (DEGs) in the cucumber virescent leaf mutant, inhibiting chlorophyll synthesis [9]. These changes in the ratio of carotenoids to chlorophyll are the main factors driving the golden leaf coloration in *Ginkgo biloba* L. mutants [10].

Chloroplast formation is a pivotal prerequisite for heterotrophic to autotrophic transformation in plants [11]. The functions of chloroplast-related genes are generally divided into three categories: transcription/translation, photosynthesis, and metabolite synthesis [12]. Leaf color mutants offer a valuable genetic resource for investigating the process of chloroplast development. The etiolated/albino appearance of the leaf color mutant *eal1* of maize is associated with changes in the levels of photosynthetic pigments and chloroplast development [13]. A pakchoi (*Brassica rapa* L. ssp. chinensis) mutant *pylm*, which has yellow leaves, also exhibits reduced total chlorophyll content and impaired chloroplast development [14]. A spontaneous *B. napus* (rapeseed) mutant *ytg*, which shows a delayed greening phenotype and retarded growth, was found to express *BnaA02.YTG1*, which encoded a chloroplast-localized tetratricopeptide repeat protein that participated in chloroplast RNA editing events [15].

Leaf color mutants are ideal reference materials for studying chloroplast structure and chlorophyll metabolism. Maintaining a normal chlorophyll level and a functioning chloroplast structure are essential for photosynthetic efficiency in plants [16], which directly influences plant growth and development [17,18]. Proteomic analysis of yellow and green *G. biloba* leaves can help identify the differentially expressed proteins related to energy metabolism, photosynthesis, and carbon fixation [19]. Over the past few decades, yellow leaf mutants of various crops, such as cucumber [7,20], maize [21], rice [22], wheat [23,24], and pakchoi [25], have been identified. Eggplant (*Solanum melongena* L.) is a popular vegetable crop, especially in Asia, Africa, the Mediterranean coast, and south-central Europe. The yellow leaf mutant *chl861-2* of eggplant has been found to exhibit significantly lower levels of total chlorophyll, chlorophyll a (Chl *a*), and chlorophyll b (Chl *b*) and net photosynthetic rate (Pn) than the wild-type (WT) plant [26]. However, the molecular mechanisms underlying the leaf color mutations in eggplant are not yet well known. In the present study, we obtained a stable heritable yellow leaf mutant line *yl20* through multi-generation inbreeding of eggplant. Our analyses revealed that a recessive nuclear gene regulated the leaf color in the mutant line.

To elucidate the mechanisms underlying leaf color formation and variation in eggplant, we analyzed and compared the cytological, physiological, and transcriptomic characters of the mutant and WT lines. Our results of the comparative analysis of the chloroplast structure, chlorophyll and carotenoid content, and transcriptomic data of mutant and WT lines can be used as a reference to further elucidate the mechanisms underlying leaf color formation in eggplant, laying a theoretical foundation for crop breeding. 

## 2. Materials and Methods

### 2.1. Plant Material

**Figure 1:**
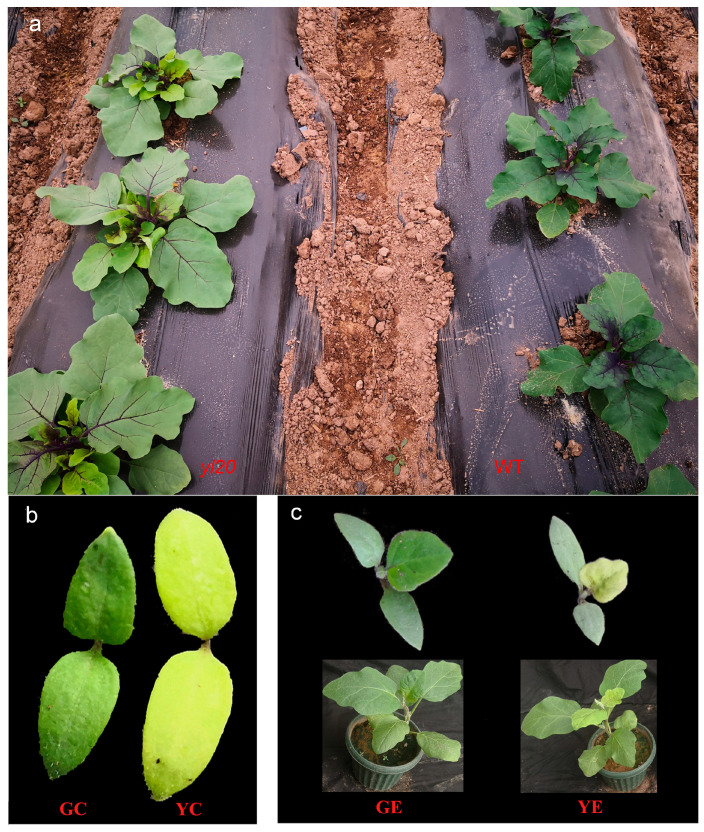
Phenotypes of the leaf color mutant *yl20* and the WT plant. (**a**) Field phenotype. (**b**) Cotyledon stage. (**c**) euphylla stage. YC, yellow leaves of *yl20* in the cotyledon stage. YE, yellow young leaves of *yl20* in the euphylla stage. GC, normal green leaves of WT in cotyledon stage. GE, normal green leaves of WT in the euphylla stage.

The yellow leaf mutant *yl20* line (Y) is a spontaneous leaf color mutant obtained from the green leaf WT line (G) of eggplant. In 2020, several yellow leaf mutants were found in homologous green leaf lines, and then the mutants were self-crossbred to obtain a homozygous yellow leaf line. This mutant was developed by the Institute of Cash Crops at the Hebei Academy of Agriculture and Forestry Sciences. The mutant plants exhibited yellow leaves in the cotyledon stage (YC), but two leaf color phenotypes (yellow young leaves and light-green mature leaves) in the euphylla stage (the young leaf (yellow) was designated YE) (Figure 1). The WT plants harbored green leaves in both the cotyledon (GC) and euphylla stages (the young leaf (green) was designated GE). The seeds were planted in seedling trays in a greenhouse on 4 January 2022. Later, 5–6 seedlings in the euphylla stage were transplanted into a plastic tunnel. We collected leaf samples of GC, YC, GE and YE from *yl20* and WT, respectively, to research the physiological characteristics, chloroplast structure, and transcriptome sequencing analysis. All samples were collected at 9 am, transferred immediately frozen in liquid nitrogen, and stored at −80 °C until use.

### 2.2. Transmission Electron Microscopy 

The dissected leaf samples from the *yl20* and WT lines were cut into smaller sections, approximately 1.0 mm × 1.0 mm × 1.0 mm in size. The samples were fixed in 4% glutaraldehyde for 24 h at 4 °C, washed thrice with 0.1 M phosphate buffer for 15 min each time, and fixed in 1% OsO_4_ for 7 h. Then, the samples were washed thrice with 0.1 M phosphate buffer for 15 min each time. Then, the samples were dehydrated, embedded, and polymerized [27]. For ultrastructural observations, 60 nm thick sections were cut using a Leica EMUC7 ultramicrotome (Leica Microsystems GmbH, Wentzler, Germany) and stained. Finally, the chloroplast ultrastructure was observed and photographed using a HITACHI HT7800 transmission electron microscope (HITACHI, Tokyo, Japan).

### 2.3. Biological and Physiological Characteristics 

The number of leaves, leaf area, stem diameter, plant height, root length, plant weight and root weight were measured in the GE and YE. The seedlings were obtained 60 days after sowing, and 20 seedlings of each sample were measured. Leaf area was measured by a handheld laser leaf area meter (CI-203, CID Instruments Co., Ltd., Camas, WA, USA). Stem diameter was measured by a vernier caliper.

The chlorophyll and carotenoid contents of leaves were measured in the GC, YC, GE and YE. The leaf samples were obtained at 30 and 90 days after sowing. For chlorophyll extraction, 0.3 g fresh leaf samples were extracted using previously described methods [25]. The supernatants were collected to assess Chl *a*, Chl *b*, total chlorophyll, and carotenoid contents using an ultraviolet-visible spectrophotometer (UV-7500, Shanghai MAPADA Instruments Co., Ltd., Shanghai, China). The Pn of the third true leaf in the four euphylla stage of *yl20* and WT were measured using a portable photosynthesis meter (LSPro-SD, ADC BioScientific Ltd., Hoddesdon, UK) in 10:00 am [28].

### 2.4. RNA Extraction and Transcriptome Sequencing 

Total RNA was extracted from each sample using the DP441 Kit (Tiangen, Beijing, China), following the manufacturer’s instructions. The RNA was sequenced using the Illumina NovaSeq 6000 high-throughput sequencing platform (Illumina, San Diego, CA, USA) available at the Shanghai Majorbio Bio-pharm Technology Co., Ltd. (Shanghai, China).

### 2.5. Transcriptome and Differential Gene Expression Analyses 

Raw reads were first filtered to obtain clean reads, which were then aligned to the eggplant reference genome (http://eggplant-hq.cn/Eggplant/home/index (accessed on 6 May 2022)) using TopHat software (Version V2.1.1) [29]. A given gene’s expression level was analyzed using the RSEM software (Version V1.3.3) and estimated by the transcripts per million reads (TPM) method. PCA analysis was obtained using the sklearn package in Python. The DEGs were analyzed using the DESeq2 software (Version 1.24.0) [30]. Transcripts that met the threshold criteria of |log2 (fold-change)| > 2 and *q*-value < 0.05 were considered DEGs. Heatmaps were obtained using the astcluster package in R. GO analysis were obtained using Goatools software (Version 0.6.5), and the Kyoto Encyclopedia of Genes and Genomes (KEGG) enrichment was performed using KOBAS software (Version 2.1.1). The GO terms and KEGG pathways were considered significantly enriched if corrected *p* (*p*-adjust) < 0.05. Volcano plot map, Venn diagram analysis and network analysis were conducted using the online platform of Majorbio Cloud. Platform (https://www.majorbio.com) (accessed on 18 May 2022).

### 2.6. Real-Time Quantitative PCR

The total RNA of leaves was extracted using Trizol and detected by 1% agarose gel electrophoresis. Reverse transcription was performed using the HiScript Q RT SuperMix for qPCR (Vazyme, Nanjing, China). Gene-specific primers were designed using Primer Premier V5.0 and actin as the reference gene (Supplementary Table S1). Then, quantitative real-time PCR (qRT-PCR) was performed using an ABI 7500 Real-Time PCR system (Applied Biosystems, Waltham, MA, USA) with the SYBR Green Supermix (Vazyme Biotech, Nanjing, China). The qRT-PCR conditions were set as follows: 95 °C for 5 min, followed by 40 cycles of 95 °C for 5 s, 50 °C for 30 s, and 72 °C for 40 s.

### 2.7. Statistical Analysis

Statistical analyses were conducted using SPSS (IBM SPSS Statistics ver.25.0; IBM Corp., Armonk, NY, USA). Duncan’s multiple range test was conducted to compare the differences between means. Differences with *p* < 0.05 were considered statistically significant. Relative expression levels of genes were calculated using the 2^−∆∆Ct^ method [31]. All data were averaged over three replicates.

## 3. Results

### 3.1. Phenotypic Characteristics

The naturally occurring *yl20* mutant of eggplant exhibited yellow YC and YE. However, at later stages of growth and development, the leaves gradually turned green. In contrast, the WT line had normal green GC and GE (Figure 1).

### 3.2. Ultrastructure Observation

**Figure 2:**
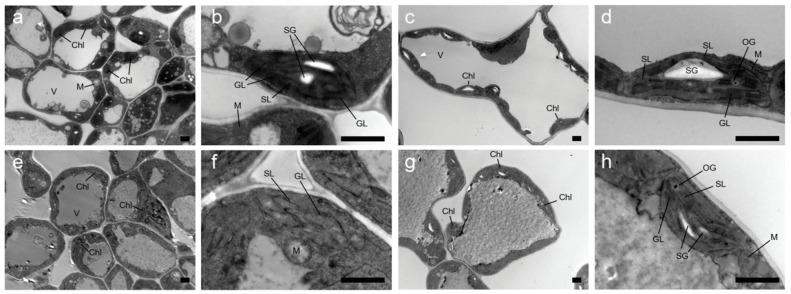
The ultrastructure of chloroplasts from the leaf color mutant *yl20* and WT. Chloroplasts ultrastructure of leaf at young leaf (**a**,**b**,**e**,**f**) and mature leaf (**c**,**d**,**g**,**h**) in WT (**a**–**d**) and *yl2*0 (**e**–**h**). Bar = 20 μm Chl: chloroplast, V: vacuole, SG: starch grain, GL: granum thylakoid, SL: stromal lamella, OG: osmophilic gramules, M: mitochondria.

We compared the chloroplast ultrastructure of the green leaves of WT lines and the yellow leaves of *yl20* lines. The green leaves (Figure 2a–d) exhibited a lower degree of damage, with complete cell structure, no plasmolysis, complete chloroplast structure, a small amount of starch grains intracellularly, and regular arrangement of grana lamellae. In contrast, the young yellow leaves (Figure 2e,f) exhibited a relatively higher degree of damage, with chloroplasts almost disintegrated, damaged and blurred membrane structure, disorganized lamellar structure, grana almost disappeared, and slightly expanded lamellar structure. The mature yellow leaves exhibited a higher degree of damage (Figure 2g,h), with damaged membrane structure, uneven and irregularly arranged grana lamella, and almost absent grana.

### 3.3. Biological and Physiological Characteristics 

Seven different biological trait parameters were detected between GE and YE. The results showed that the stem diameter, plant height, plant weight, and root weight levels of YE were significantly lower than those of GE. The leaf number and leaf area levels were significantly higher than those of GE (Table S2).

The physiological datasets (Table 1) showed that the mutant samples exhibited significantly lower levels of total chlorophyll, Chl *a*, Chl *b*, and carotenoids than the WT samples in both cotyledon and euphylla stages. The Chl *a*/Chl *b* ratio was higher in the mutant samples in the cotyledon stage, but the ratio was higher in the WT samples in the euphylla stage. The mutant samples exhibited a lower chlorophyll/carotenoid ratio than the WT samples at all stages.

### 3.4. Transcriptome Analysis 

A total of 12 RNA samples from the WT and mutant leaves in the cotyledon and euphylla stages were sequenced. After removing the low-quality reads, a total of 599,789,020 clean reads were obtained and mapped to the reference genome of eggplant (Supplementary Table S3). The Q20 and Q30 percentage values were >90%, and the mapping ratio was >96% (Supplementary Table S3). The principal component analysis indicated that the three biological repeats for each of the 12 samples were relatively clustered in the ordinal space (Supplementary Figure S1). The first (PC1) and the second principal components (PC2) accounted for 56.94% and 15.79% of the variation in the data, respectively. Taken together, these findings confirmed that the data were reliable and suitable for subsequent analyses.

### 3.5. Identification of DEGs 

**Figure 3:**
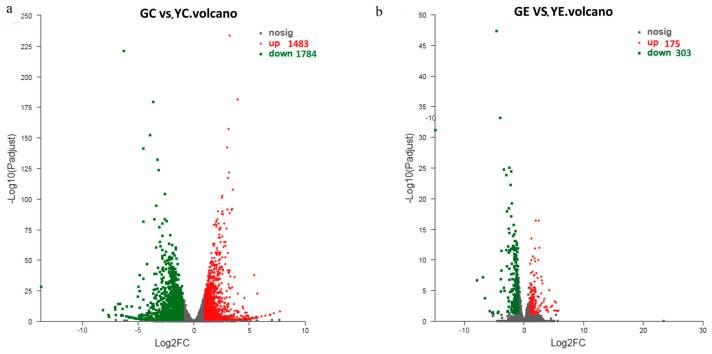
Volcano plot map of differentially expressed genes (DEGs) in the leaf color mutant *yl20* and the WT. (**a**) The DEGs number in the cotyledon stage. (**b**) The DEGs number in the euphylla stage. YC, yellow leaves of *yl20* in the cotyledon stage. YE, yellow young leaves of *yl20* in the euphylla stage. GC, normal green leaves of WT in cotyledon stage. GE, normal green leaves of WT in the euphylla stage.

To assess the variations in gene expression, we used the TPM values to identify DEGs. We assessed the DEGs among the four groups: GC, GE, YC, and YE. We detected 3267 DEGs between the GC and YC groups (the GC and YC groups were the contrast and test groups, respectively). Between the GE and YE groups, 478 DEGs were identified (Figure 3). Thus, compared to the cotyledon stage, significantly fewer DEGs (14.63% of those in the cotyledon stage) were identified in the euphylla stage. Moreover, most DEGs were downregulated in the mutant samples in both cotyledon and euphylla stages (Figure 3).

### 3.6. GO Functional Analysis

**Figure 4:**
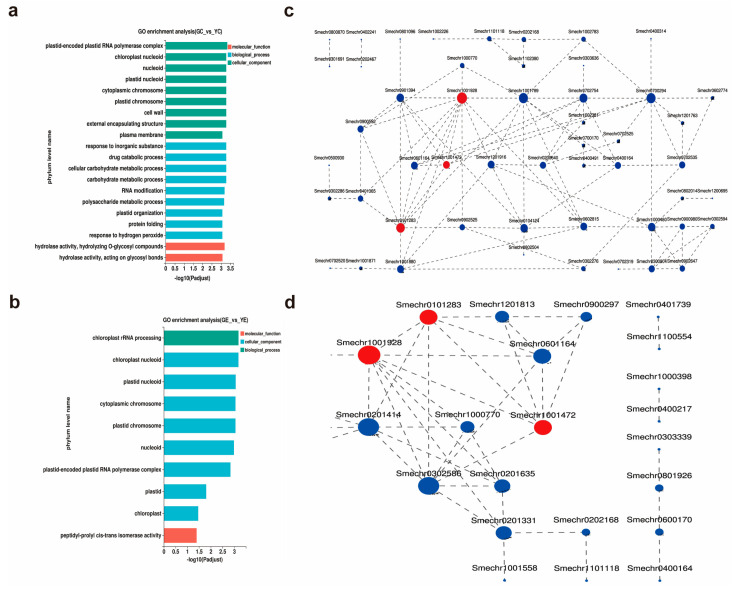
GO classification and PPI network of DEGs in the leaf color mutant *yl20* and the WT. (**a**) Analysis of GO terms enriched for the 3267 DEGs in the cotyledon stage. (**b**) Analysis of GO terms enriched for the 478 DEGs in the euphylla stage. (**c**) Analysis of PPI networks of the 74 DEGs associated with chloroplast in the cotyledon stage. (**d**) Analysis of PPI networks of the 43 DEGs associated with chloroplast in the euphylla stage. YC, yellow leaves of *yl20* in the cotyledon stage. In the constructed networks, the size of a circle indicated the significance of the corresponding gene, with larger circles representing more important genes. Red circles represent the common DEGs between cotyledon and euphylla stages, and the blue circles represent the unique genes of the respective stages. YE, yellow young leaves of *yl20* in the euphylla stage. GC, normal green leaves of WT in cotyledon stage. GE, normal green leaves of WT in the euphylla stage.

Next, the identified DEGs were subjected to GO enrichment analysis. The biological functions of the DEGs were classified according to the GO database. In the cotyledon stage, a total of 79 GO terms were enriched (Supplementary Table S4), which were then divided into three categories: Biological process (BP; 41.1%), molecular function (MF; 48.7%), and cellular component (CC; 10.2%). The top 20 terms that were significantly enriched are listed in Figure 4a. The “plastid-encoded plastid RNA polymerase complex” (GO:0000427; *p*-adjust = 0.000462) was the most significant term. In the euphylla stage, the DEGs were enriched in 10 terms spanning the three categories: BP (4.10%), MF (5.74%), and CC (90.16%) (Figure 4b). The “chloroplast rRNA processing” (GO:1901259; *p*-adjust = 0.000666) and “chloroplast nucleoid” (GO:0042644; *p*-adjust = 0.000666) were the most significant terms.

Since cytological observations revealed marked chloroplast damage in the mutant, we focused on the chloroplast-related GO terms. Among the 79 terms enriched in the cotyledon stage, four terms, comprising 74 genes, were related to chloroplast: “chloroplast nucleoid”, “chloroplast organization”, “chloroplast stroma”, and “chloroplast rRNA processing”. Of these 10 terms enriched in the euphylla stage, three terms, comprising 43 genes, were related to chloroplast: “chloroplast rRNA processing”, “chloroplast nucleoid”, and “chloroplast”. To further explore the gene interaction and identify the chloroplast-related core genes, expression interaction (protein–protein interaction (PPI)) networks of the 74 and 43 DEGs associated with chloroplast development were constructed. The PPI networks showed *Smechr1001928* (*Fln2*), *Smechr0101283* (*CITRX*), and *Smechr1001472* (*SODF2*) as common DEGs between the cotyledon and euphylla stages. Moreover, these three DEGs were significantly downregulated in the mutant than WT in both growth stages (Supplementary Table S5). Of these three DEGs, the circle associated with *Smechr1001928* was the largest in both growth stages, indicating that it might play a key role in yellow leaf formation (Figure 4c,d).

### 3.7. KEGG Functional Analysis 

**Figure 5:**
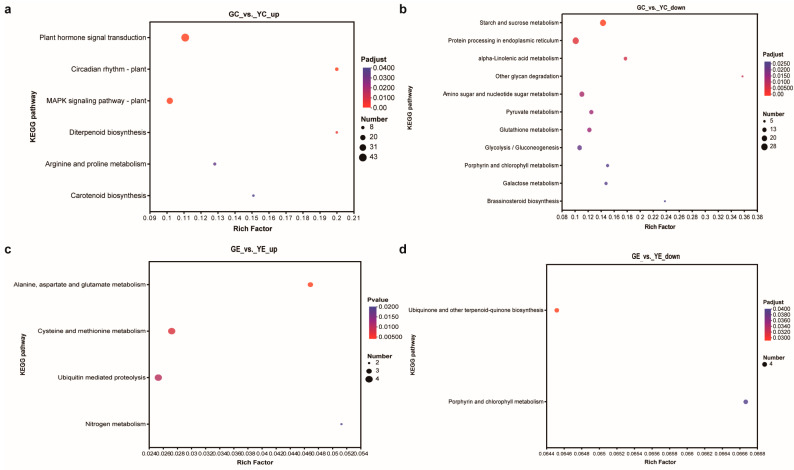
The KEGG pathways enrichment analysis of DEGs in the leaf color mutant *yl20* and the WT. (**a**) Analysis of KEGG pathways enriched for the upregulated DEGs in the cotyledon stage. (**b**) Analysis of KEGG pathways enriched for the downregulated DEGs in the cotyledon stage. (**c**) Analysis of KEGG pathways enriched for the upregulated DEGs in the euphylla stage. (**d**) Analysis of KEGG pathways enriched for the downregulated DEGs in the euphylla stage. YC, yellow leaves of *yl20* in the cotyledon stage. YE, yellow young leaves of *yl20* in the euphylla stage. GC, normal green leaves of WT in cotyledon stage. GE, normal green leaves of WT in the euphylla stage.

**Figure 6:**
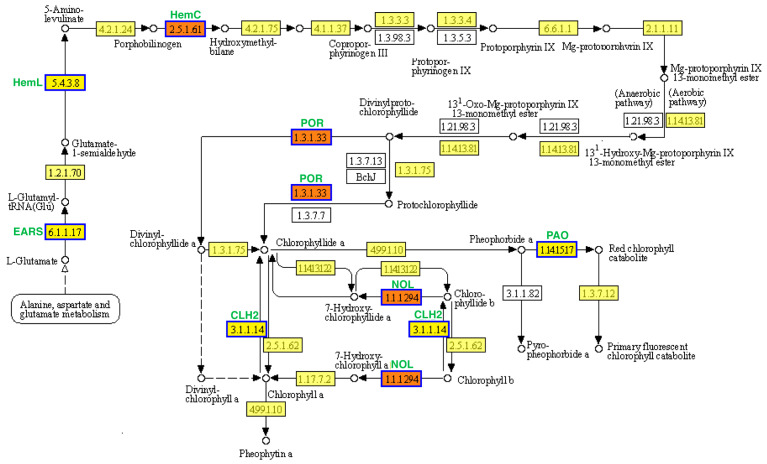
Porphyrin and chlorophyll metabolism pathway analysis of DEGs in the leaf color mutant *yl20* and the WT. Yellow boxes represents annotated genes, white boxes represent unknown genes, and red boxes represent the genes involved in both cotyledom and euphylla stages. The solid arrows represent the direct action and the dashed arrows represent the indirect action.

KEGG analysis was used to identify the enriched biological pathways. In the cotyledon stage, the upregulated and downregulated DEGs were significantly enriched in 6 and 11 pathways, respectively (Figure 5a,b). In the euphylla stage, the upregulated and downregulated DEGs were significantly enriched in four and two pathways, respectively (Figure 5a,b). The “porphyrin and chlorophyll metabolism” pathway was common between both growth stages, indicating that this pathway might be crucially related to leaf color mutation. Furthermore, nine and four DEGs in the cotyledon and euphylla stages, respectively, were associated with this pathway. Of these, three DEGs, *Smechr0400217* (*POR*), *Smechr0702754* (*HemC*), and *Smechr100398* (*POR*), were common in both growth stages (Figure 6, Table 2).

### 3.8. Venn Analysis

**Figure 7:**
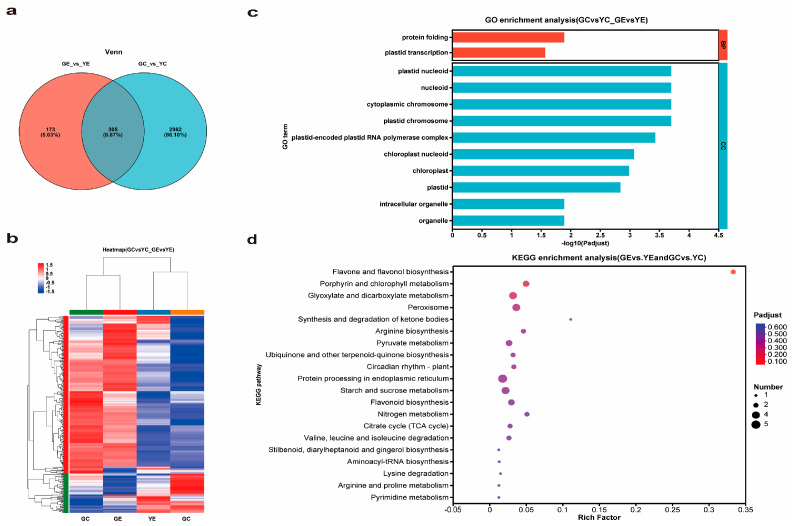
DEGs and function analysis. (**a**) Venn diagram analysis of DEGs. (**b**) Heat map analysis of 305 DEGs. (**c**) GO analysis of 305 DEGs. (**d**) KEGG analysis of 305 DEGs.

Venn diagram analysis of the two group DEGs (Figure 7a) showed that 305 DEGs were in common between GE_vs._YE and GC_vs._YC. The heat map showed that most of those DEGs underwent downregulated expression in *yl20* compared with WT (Figure 7b). By analyzing the 305 DEGs, we found 12 significantly enriched GO terms, and 10 of which were contained in CC and two in BP (Figure 7c, Supplementary Table S6). Further analysis of these terms revealed that 5 were related to plastids and 2 chloroplasts. KEGG analysis of the 305 DEGs indicated their relevance to 58 pathways in total. The top two significantly enriched pathways were ‘flavone and flavonol biosynthesis’ and ‘porphyrin and chlorophyll metabolism’ (Figure 7d, Supplementary Table S7).

### 3.9. qRT-PCR

To validate the reliability of transcriptomic sequencing data, we performed qRT-PCR analysis of the identified DEGs related to chlorophyll and chloroplast. The qRT-PCR results aligned well with the RNA-sequencing results (Supplementary Figure S2).

## 4. Discussion

Leaf color development is a complex and sensitive process regulated by various genes and metabolic pathways, such as chlorophyll biosynthesis and degradation, carotenoid synthesis and degradation, chloroplast development [32], and photosynthesis [33]. Leaf color mutants serve as excellent models for investigating the underlying mechanisms of leaf color alter. Though several studies have focused on the leaf color development process, the mechanisms underlying leaf color development in plants remain unclear.

### 4.1. Chlorophyll Metabolism

Chlorophyll synthesis is a highly regulated process, and several chlorophyll metabolism models have been established. Being the key photosynthetic pigment in plants, changes in chlorophyll content lead to substantial leaf color development [34]. In the present study, we detected significantly lower chlorophyll and carotenoid contents in yellow leaves than in green leaves, underscoring the phenotypic variation between WT and *yl20* lines.

In plants, chlorophyll (Chl *a* plus Chl *b*) is potentially the most abundant and important tetrapyrrole [35]. Research has shown that the chlorophyll synthesis process comprises 16 steps, beginning with glutamyl-tRNA (Glu-tRNA) and ending with Chl *b*. It has been found that this process requires 16 enzymes encoded by more than 20 genes [36]. In *Arabidopsis*, chlorophyll synthesis is mediated by 15 enzymes encoded by 27 genes. Any alterations in the expression profile of these genes might lead to chlorophyll metabolic disorders and a yellow leaf phenotype in plants [37]. In the present study, we divided the chlorophyll synthesis process into seven key steps. The first step was the conversion of Glu-tRNA to δ-Aminolevulinic acid (ALA), and the second step was the conversion of ALA to porphyrinogen III. These reactions occur in anaerobic conditions. The third step was the conversion of porphyrinogen III to protoporphyrin IX. In the fourth step, protoporphyrin IX receives magnesium (Mg) ions and forms Mg-protoporphyrin. The fifth step was the conversion of Mg-protoporphyrin to protochlorophylide a. The sixth step was the conversion of protochlorophylide a to chlorophylide a. The final step involved the transformation of chlorophylide a into Chl *a*, which is interconverible with Chl *b*.

Previous studies have found that suppressing porphobilinogen deaminases (HemC) expression in transgenic tobacco leads to reduced urinary porphyrin 3 (PBGD) activity, restricting the transformation of PBG into Urogen III, resulting in a decreased chlorophyll content and light-green color of leaves [38]. Protochlorophyllide oxidoreductase (POR) is a light-dependent enzyme involved in chlorophyll biosynthesis and is essential for photosynthesis [2]. POR regulates the greening process during the development of heterotrophic cotyledons to true leaves of autotrophic seedlings. Reduced POR levels lead to delayed greening of etiolated seedlings [39]. Thus, regulation of POR expression is central to the chlorophyll biosynthetic pathway and seedling greening. For instance, POR overexpression leads to a higher chlorophyll content in transgenic tobacco [40]. Another study demonstrated that NYC1 encodes Chl *b* reductase, which catalyzes the degradation of Chl *b* to 7-hydroxymethyl Chl *a* [41]. The NYC1-like (NOL) protein is closely related to NYC1, and its overexpression drastically reduces Chl *b* levels [42,43].

In the present study, we identified nine chlorophyll metabolism-related DEGs between *yl20* and WT in the cotyledon stage, all of which were downregulated in the mutant samples. We observed a four-fold higher expression of chlorophyll biosynthesis gene *POR* and the chlorophyllase gene *CLH* in green leaves than in yellow leaves in the cotyledon stage. Furthermore, in the euphylla stage, we identified four chlorophyll metabolism-related DEGs, all of which were downregulated in the mutant line. Previous studies have shown that the *CLH1* and *CLH2* genes in *Arabidopsis* are highly homologous to the *CLH* genes in eggplant. Notably, the deletion of *CLH1* and *CLH2* in *Arabidopsis* does not impact chlorophyll degradation. These findings indicated that downregulation of chlorophyll biosynthesis genes hinders chlorophyll synthesis. Thus, the lower chlorophyll content in the *yl20* might be attributed to impaired expression of these genes. Moreover, decreasing total chlorophyll content results in relative elevation of carotenoid content. In addition, *NOL/NYL* downregulation leads to restricted conversion of Chl *b* to Chl *a*, resulting in a relative increase in Chl *b* content. Taken together, the relatively high carotenoid and Chl *b* contents in the *yl20* mutant might be responsible for its yellow leaf phenotype.

### 4.2. Chloroplast Development 

When seedlings are exposed to light, chlorophyll synthesis commences, and the cotyledons turn green [44]. Meanwhile, the etioplasts of cotyledons develop into chloroplasts, enabling the seedlings to become photoautotrophic [45]. Eventually, plant seedlings develop morphological traits required for photosynthesis in the presence of incident light, such as chloroplast-rich cotyledons and shortened hypocotyls. This mode of development is termed photomorphogenesis [46]. In leaves, starch is formed in the chloroplasts during the day from photo-assimilated CO_2_ [47]. Disrupted chloroplast function often leads to a severe phenotype, such as albino or pale green [48]. By observing the chloroplast ultrastructure, we found that the chloroplast was apoptotic in *yl20*. Through transcriptome analysis, we found many genes related to cell component of chloroplast were downregulated in *yl20*. Then we speculate that the yellow leaf formation was possibility produced by a reduction in the synthesis of proteins able to form the lipoprotein complex present in the chloroplast.

Chloroplast transcription machinery is complex and primarily regulates chloroplast development [49]. Generally, chloroplast-encoded genes are transcribed by either plastid-encoded polymerase (PEP) or nuclear-encoded polymerase (NEP) [11]. In a previous study, a set of maize (*Zea mays*) mutants lacking PEP-associated proteins were found to exhibit similar ivory/virescent pigmentation and corresponding reductions in their plastid ribosomes and photosynthetic complexes [50]. The DEGs identified in the present study were also enriched to PEP, the photomorphogenesis term, and starch and sucrose metabolism in the cotyledon stage. These genes were downregulated in the *yl20* mutant, which might have led to the yellow leaf formation in the cotyledon.

Fructokinase-like proteins (FLNs) are phosphofructokinase-B (PfkB)-type carbohydrate kinases that act as part of the PEP complex [51]. Thioredoxinz (TRXz), regulates PEP-dependent chloroplast transcription and is essential for proper chloroplast development [52]. OsFLN1 and HSA1/OsFLN2 interact with rice TRXz (OsTRXz) to regulate chloroplast development, and OsTRXz knockout resulted in an albino phenotype similar to that of *fln1* mutants [53]. In *Arabidopsis*, the *fln2–4* mutant [54] is similar to other group of delayed greening mutants, such as *YS1* [55] and *dg1* [56]. Previous studies have shown that, on sucrose-containing medium, the *fln2–4* mutant in *Arabidopsis*, which usually displays an albino phenotype, can develop greenish true leaves, while the *fln2–1* and *fln2–2* mutants still exhibited pale-green cotyledons and delayed greening [50]. The *fln* mutants exhibit severe phenotypes [50]. The *fln1* mutants exhibit an albino phenotype. In contrast, the *fln2* plants display chlorosis prior to leaf expansion but undergo delayed greening, remain autotrophic, can grow to maturity, and provide viable seeds [50]. As the *fln2–4* mutant retains partially PEP activity, exogenous sucrose application leads to the development of the plastids in the *fln2* mutants into fully functional chloroplasts, enabling them to eventually develop the green phenotype [50]. In the present study, abnormal chloroplast structure was detected in the yellow leaf mutant. Moreover, we identified *Fln2* as one of the DEGs between the mutant and WT lines. This gene interacts with other genes and impacts chloroplast development. *Fln2* was downregulated in *yl20*, potentially contributing to the yellow color leaf and delayed plant growth, similar to the *fln2* mutant of *Arabidopsis* [50]. Thereby, *Fln2* might be crucially involved in the leaf color development in the *yl20* mutant. Our findings provided novel insights into the molecular mechanisms underlying leaf color regulation in eggplants.

## Figures and Tables

**Table 1:** Comparison of chlorophyll and carotenoid contents of leaves in the leaf color mutant *yl20* and the WT.

Sample	Chl *a* (mg·g^−1^)	Chl *b* (mg·g^−1^)	Total Chlorophyll (mg·g^−1^)	Carotenoid (mg·g^−1^)	Chl *a*/Chl *b*	Chlorophyll/Carotenoid	Pn (μmol CO_2_·m^−2^·s^−1^)
GC	0.76 ± 0.01 a	0.25 ± 0.01 a	1.01 ± 0.02 a	0.27 ± 0.01 a	3.05 ± 0.02 b	3.74 ± 0.01 a	-
YC	0.34 ± 0.00 b	0.07 ± 0.00 b	0.40 ± 0.02 b	0.11 ± 0.00 b	4.83 ± 0.09 a	3.49 ± 0.03 b	-
GE	1.26 ± 0.03 a	0.49 ± 0.03 a	1.75 ± 0.05 a	0.44 ± 0.01 a	2.44 ± 0.04 a	3.96 ± 0.09 a	16.62 ± 0.21 a
YE	0.56 ± 0.03 b	0.28 ± 0.02 b	0.84 ± 0.05 b	0.23 ± 0.01 b	2.02 ± 0.09 b	3.59 ± 0.07 b	13.19 ± 0.30 b

Note: Different letters denote significant differences according to Duncan’s test at the 0.05 level. The results were expressed as mean ± SE.

**Table 2:** The DEGs of ‘porphyrin and chlorophyll metabolism’ pathway in the leaf color mutant *yl20* and the WT.

Group (Number)	Gene_ID	KO_Name	YC_TPM	GC_TPM	YE_TPM	GE_TPM	Swiss-Prot_Hit-Name	Swiss-Prot_Description
GC_vs._YC	*Smechr0100006*	EARS, gltX	44.21	97.59	34.05	66.37	SYE_TOBAC	Glutamate–tRNA ligase, chloroplastic/mitochondrial
	*Smechr0400217*	por	3.19	13.25	48.64	106.34	POR_DAUCA	Protochlorophyllide reductase, chloroplastic
	*Smechr0601548*	E3.1.1.14	3.05	11.57	5.92	8.1	CLH2_ARATH	Chlorophyllase-2, chloroplastic
	*Smechr0701042*	NOL, NYC1	29.78	64.52	31.81	42.08	NYC1_ORYSJ	Probable chlorophyl
	*Smechr0702754*	hemC, HMBS	91.23	213.51	137.76	293.49	HEM3_PEA	Porphobilinogen deaminase, chloroplastic
	*Smechr1000398*	por	38.89	105.32	191.43	421.27	PORA_CUCSA	Protochlorophyllide reductase, chloroplastic
	*Smechr1000445*	E3.1.1.14	0.59	2.44	68.96	116.72	CLH1_ARATH	Chlorophyllase-1
	*Smechr1102388*	hemL	189.34	386.83	214.97	404.78	GSA_SOLLC	Glutamate-1-semialdehyde 2,1-aminomutase, chloroplastic
	*Smechr1201808*	PAO, ACD1	18.94	42.01	19.85	28.11	PAO_ARATH	Pheophorbide a oxygenase, chloroplastic
GE_vs._YE	*Smechr0800857*	NOL, NYC1	32.4	49.14	13.67	32.12	NOL_ARATH	Chlorophyll
	*Smechr0400217*	por	3.19	13.25	48.64	106.34	POR_DAUCA	Protochlorophyllide reductase, chloroplastic
	*Smechr0702754*	hemC, HMBS	91.23	213.51	137.76	293.49	HEM3_PEA	Porphobilinogen deaminase, chloroplastic
	*Smechr1000398*	por	38.89	105.32	191.43	421.27	PORA_CUCSA	Protochlorophyllide reductase, chloroplastic

## Data Availability

The mRNA sequencing data were deposited in the NCBI sequence read archive (SRA) under the accession number PRJNA793301.

## Supplementary Materials

The following supporting information can be downloaded at: https://www.mdpi.com/article/10.3390/plants13060855/s1.
