# Supplementary material for: Cytological, Physiological, and Transcriptomic Analyses of the Leaf Color Mutant Yellow Leaf 20 (yl20) in Eggplant (Solanum melongena L.)
Source: Plants (Basel). 2024 Mar 15;13(6):855. doi: 10.3390/plants13060855 (PMC10974653; doi:10.3390/plants13060855)

Supplementary Figure S1 The PCA analysis of the leaf-color mutant *yl20* and the WT in eggplant. YC, yellow leaves of *yl20* in the cotyledon stage. YE, yellow young leaves of *yl20* in the euphylla stage. GC, normal green leaves of WT in cotyledon stage. GE, normal green leaves of WT in the euphylla stage.

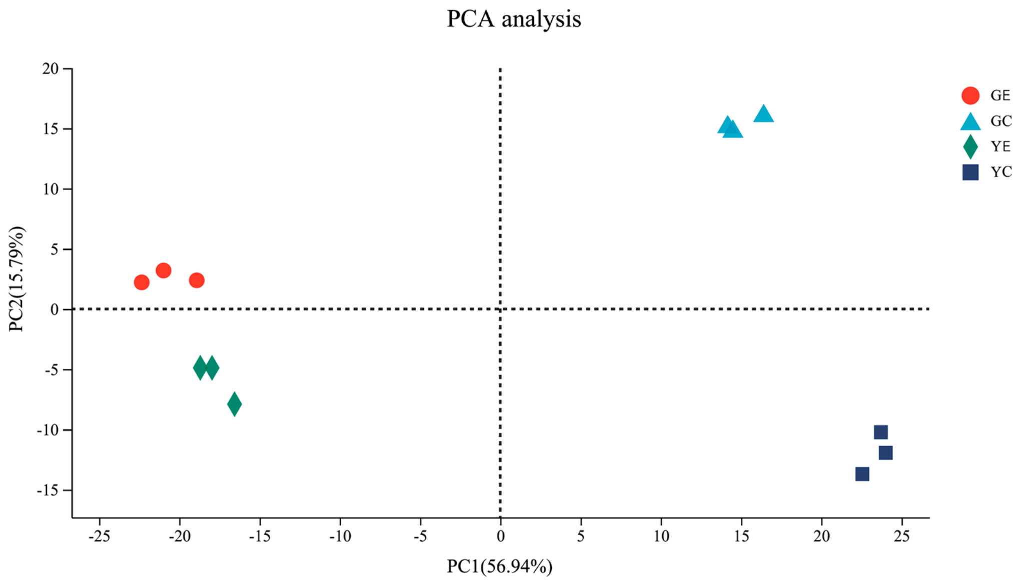

Supplementary Figure S2 qRT-PCR confirmation of DEGs identified by transcriptome analysis of the leaf-color mutant *yl20* and the WT in eggplant. YC, yellow leaves of *yl20* in the cotyledon stage. YE, yellow young leaves of *yl20* in the euphylla stage. GC, normal green leaves of WT in cotyledon stage. GE, normal green leaves of WT in the euphylla stage.

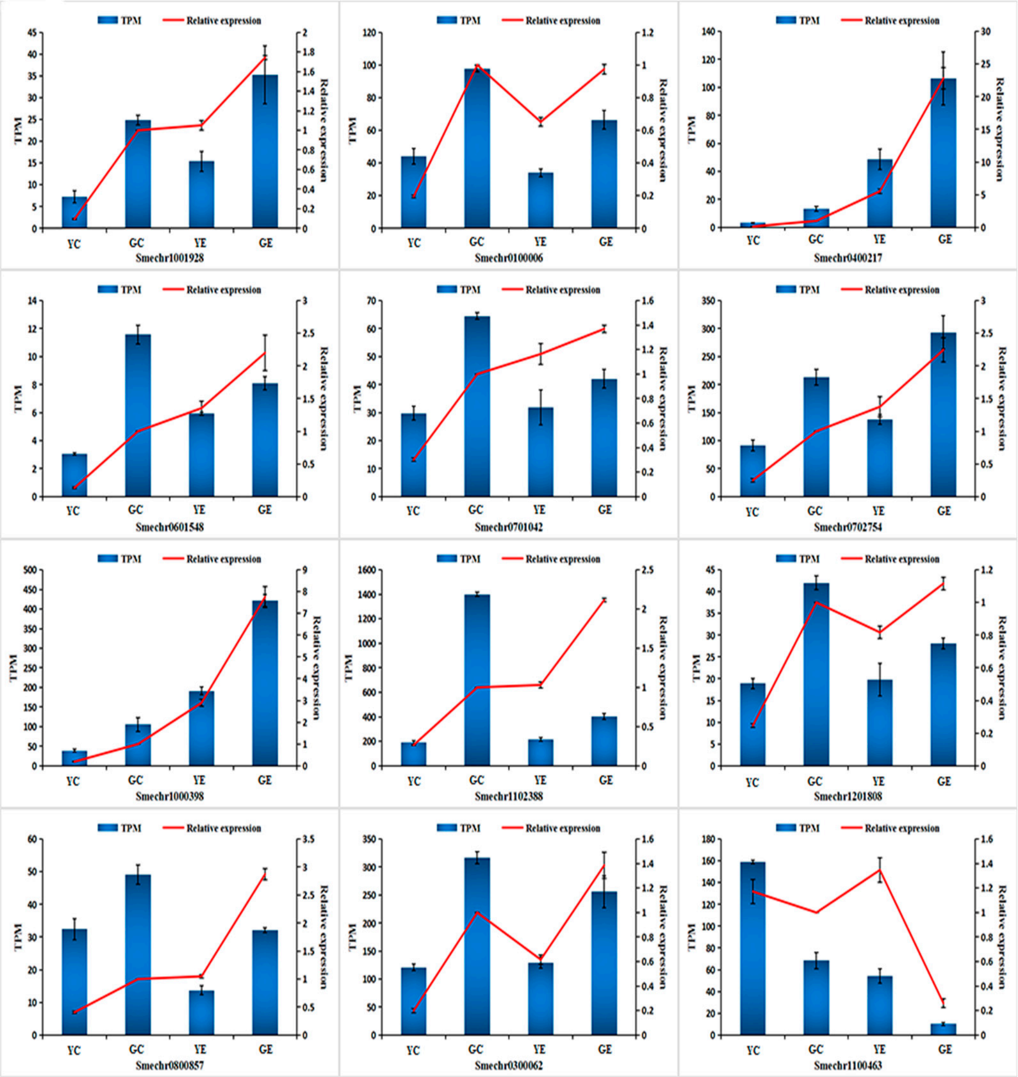

Supplement: Supplementary file 1 [file plants-13-00855-s001.zip › Supplementary Figures-Plants.pdf]
